# Tracking of autologous adipose-derived mesenchymal stromal/stem cells after intravenous administration: a pilot study in a dog with induced acute bladder injury

**DOI:** 10.3389/fvets.2025.1644746

**Published:** 2025-12-05

**Authors:** Mathilde Porato, Nadine Antoine, Olivier Waroux, Joëlle Piret, Stéphanie Noël, Annick Hamaide

**Affiliations:** 1 Clinical Department of Companion Animals, School of Veterinary Medicine, University of Liège, Liège, Belgium; 2 Department of Morphology and Pathology, School of Veterinary Medicine, University of Liège, Liège, Belgium; 3 High School Charlemagne, Liège, Belgium

**Keywords:** dog, stromal cells, autologous, intravenous, bladder injury

## Abstract

**Introduction:**

Regenerative therapy for bladder diseases has been studied in rodent to restore bladder function after a chronic and irreversible bladder wall deterioration. These studies rarely demonstrate the presence of stem cells in the bladder. Cell-tracking after intravenous (IV) administration of stem cells enables to confirm the homing potential of an injury. Our objective was to assess, in one dog, the homing capability of autologous adipose-derived mesenchymal stromal/stem cells (ADMSCs) injected intravenously to an acute bladder injury.

**Methods:**

Adipose-derived mesenchymal stromal/stem cells were isolated from the subcutaneous tissue of a dog and labelled. As a homing signal, a full-thickness bladder biopsy representing an acute injury was created in this dog (day 0, control time). Twenty million autologous PKH26-labelled ADMSCs were injected in the cephalic vein on days 1, 4 and 8. Urinalysis was performed (day 5). Bladder biopsy was repeated at the location of the previous scar to assess the presence of labelled ADMSCs in the bladder wall (day 10).

**Results:**

Labelled ADMSCs were observed in the second bladder biopsy, not in the initial biopsy nor in urine. The only adverse event mild, self-limiting hematuria. Complete cell blood count, blood urea nitrogen and plasma creatinine were within normal limits (day 5).

**Conclusion:**

The comparison of bladder biopsies before and after IV administration of autologous ADMSCs indicates that they reached the bladder injury. Our protocol was feasible and safe. Hematuria was probably due to the bladder biopsy. These results could encourage the evaluation of this protocol in larger cohorts of dogs.

## 1 Introduction

The therapeutic role of mesenchymal stem cells (MSCs) has been studied after *in situ* administration in rodent models of bladder disorders, such as chemical induced cystitis (1), interstitial cystitis/bladder pain syndrome (2), Parkinson disease (3) or spinal cord injury (4). In rats with partial bladder outlet obstruction, promising structural and functional results were obtained after intravenous (IV) injection of labelled MSCs (5). Intravenous administration of stem cells is regarded as an advantage in medical research, due to the low degree of invasiveness to obtain the therapeutic effect, compared to arterial or parenchymal injection (6). Intravenous administration also has the potential to treat systemic diseases (7). Due to filtering organs (lungs, liver, spleen), engraftment rate of MSCs after IV injection is expected to be lower than after arterial or parenchymal administration. Engraftment rate also depends on homing efficiency, MSCs survival within ischemic diseased tissues as well as timing and methods of MSCs detection (7).

To ascertain the homing of stromal/stem cells in specific tissues, including the target one, different tracking methods have been reported. Tracking methods can be tested on stromal/stem cells *in vitro* to assess their potential limitations before further experiment. Thus, stem cell loss due to labelling or different detection sensitivity and specificity have been reported (8–10). Tracking methods using imaging as an alternative to histological techniques, have been developed to avoid tissue biopsy (11, 12). In these studies, stem cells are labelled with a radiotracer 111In oxine (11) or with superparamagnetic iron oxide particles (12). More conventional tracking methods have been reported in dogs, such as the use of green fluorescent protein (GFP) (13, 14). To allow the expression of GFP by the MSCs, this technique requires introduction of the GFP gene into the cells with a vector. Other tracking techniques, like lipophilic dyes of the cell membrane, are also routinely used, such as CM-DiI (15) or PKH26 (16), and are easily detected by conventional fluorescent microscopy. Independently of the tracking method, determination of the precise timing for detection of transplanted MSCs into host tissues is challenging. In one study, intradermally transplanted goat-derived MSCs were detected at the wound site even after completion of the healing process (14 days) in a rabbit model of cutaneous wound (17).

Most studies describe the use of allogenic MSCs (18–20). While most studies report the advantages of allogenic origin of MSCs, such as immunosuppressant effect (21, 22) and immediate availability (23), other authors point out the risk for inflammatory (24) or even immune response (25, 26) and rejection (27–29), as well as for delayed disorders related to chronic graft versus host disease, such as infections, secondary cancers and organ dysfunction (30). Doubts have raised when systemic or multiple injections (31) are required or when studies report no difference between allogenic and autologous stem cells in healthy subjects (32), while other clearly show the superiority of autologous stem cells in diseased patients (33). Moreover, transplantation of allogenic MSCs represents a risk for transmission of infectious diseases. These potential issues have led us to consider the use of autologous MSCs.

It is widely accepted that transplanted MSCs do not contribute to tissue repair through differentiation but rather through paracrine effect on host cells (34–36). In an *in vitro* study, it has been demonstrated that canine adipose-derived mesenchymal stem cells secrete cytokines and growth factors, the most secreted component being the Monocyte Chemoattractant Protein-1 (37).

Knowledge about canine autologous ADMSCs is limited. Our study was designed to obtain labelled autologous ADMSCs from one dog and to assess their homing capability following IV injection in the same dog after induction of acute bladder inflammation with a bladder biopsy. At this point, tissue regenerative potential supported by ADMSCs was beyond the scope of the present study phase.

We hypothesized that canine autologous labelled ADMSCs could be obtained and that these ADMSCs injected through IV route would reach an inflamed site, without critical adverse effect on the host. We also hypothesized that the ADMSCs would survive within the targeted host tissue throughout a healing period of 10 days after their administration.

## 2 Materials and methods

### 2.1 *In vitro* study

#### 2.1.1 Isolation, culture and characterization of ADMSCs

Adherent and fibroblast-like cells were obtained from subcutaneous adipose tissue explants of a dog as previously described (38). These cells were characterized in order to satisfy the minimal criteria of the International Society for Cell Therapy (39, 40) which have already been described in dogs (41, 42). Briefly, cells at their third passage were incubated with antibodies anti-CD29 [phycoerythrin conjugated mouse monoclonal IgG1, BioLegend (San Diego, CA, USA)], anti-CD44 [phycoerythrin conjugated mouse monoclonal IgG1, BioLegend (San Diego, CA, USA)], anti-CD90 [phycoerythrin conjugated rat monoclonal IgG2b kappa, eBioscience, Thermofischer Scientific, (Waltham, MA, USA)] and anti-CD45 [fluorescein conjugated rat monoclonal IgG2b kappa, eBioscience, Thermofischer Scientific, (Waltham, MA, USA)]. After 1 h, cells were rinsed before analyze with flow cytometry.

In addition, the cells multipotency was assessed by their ability to differentiate into adipogenic, chondrogenic and osteogenic cells. Briefly, differentiation was induced according to manufacturer’s guidelines [Stempro differentiation kits, Gibco, ThermoFischer Scientific (Waltham, MA, USA)] and following specific periods of culture (respectively 7, 14 and 21 days) in appropriate differentiation media. Ultimately, adipogenic differentiation was checked by Oil Red O staining (Sigma-Aldrich) for the presence of lipid droplets in the cytoplasm. The osteogenic differentiation was assessed by Alizarin Red S staining for calcium deposits in extracellular matrix (Sigma-Aldrich). For chondrogenic differentiation, the proteoglycan-rich extracellular matrix was detected by Alcian Blue staining.

After a fourth passage, aliquots of these cells were prepared and stored in liquid nitrogen for subsequent use.

#### 2.1.2 Stromal/stem cell labelling

**Figure 1:**
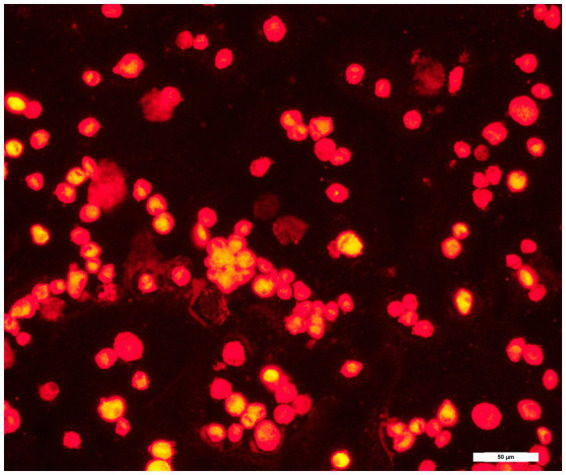
Red fluorescence of the PKH26-labelled ADMSCs *in vitro*, immediately after staining.

ADMSCs were labelled with a red fluorescent membrane dye, PKH26, at low passage (less than 6) according to manufacturer recommendations [PKH26 Red Fluorescent cell Linker Kit, obtained from Sigma Aldrich (Saint Louis, MI, USA)]. Briefly, frozen cells were thawed, cultured until 90% confluence in 2 flasks T175, and then trypsinized. The detached ADMSCs were at passage 5 and were washed by a serum-free medium and resuspended in 1 mL of dilution buffer from the manufacturer’s labelling kit. The cell suspension was mixed with an equal volume of the labelling solution containing 4.10^−6^ M of PKH26 in the dilution buffer and incubated for 5 min at room temperature. After the termination of the reaction by adding 2 mL fetal bovine serum [Gibco, ThermoFischer Scientific (Waltham, MA, USA)], cells were washed 3 times with the Dulbecco’s modified Eagle’s medium [Gibco, ThermoFischer Scientific (Waltham, MA, USA)] and observed by fluorescent microscopy (Figure 1). A fraction of PKH26 labelled cells was maintained in culture during 10 days to monitor their long-term stability and viability.

#### 2.1.3 Assessment of viability

To ensure a viability rate superior to 90% before injection, living cells were counted after a Trypan blue staining [Invitrogen, ThermoFischer Scientific (Waltham, MA, USA)]. Viability was assessed twice: after cryopreservation and labelling, and for labelled cells, after 10 days in culture medium.

### 2.2 *In vivo* study

#### 2.2.1 Dog

A healthy intact male Beagle dog of 1 year of age and weighing 13 kg was used for the study as both the donor and recipient of the ADMSCs. He was born and housed at the animal facilities of the Faculty of Veterinary Medicine of the University of Liège. Animal housing, care, and experimental procedures involved in this work were approved by the Ethical Committee of Animal Use of the University of Liège (approval number 17–1924). The dog had no clinical sign of urinary tract disease and complete urinalysis was normal. Prior to each experiment, a complete physical examination of the dog was performed. After each biopsy, the dog was monitored for 2 days (appetite, micturition, physical examination) and buprenorphine (Dechra, England, 15 μg/kg, IV) was given every 6 h. Urinalysis included a dipstick test, specific gravity measurement and cytologic examination. Eight weeks prior to ADMSCs administration, a subcutaneous adipose tissue biopsy was surgically obtained from the ventral abdominal region under general anesthesia. As a premedication, the dog received methadone (0.3 mg/kg IV) and midazolam (0.2 mg/kg IV). Anesthesia was induced with a bolus of propofol (2 mg/kg IV) and maintained with isoflurane in oxygen after placement of a cuffed endotracheal tube.

#### 2.2.2 Creation of an acute bladder injury and ADMSCs administration

**Figure 2:**
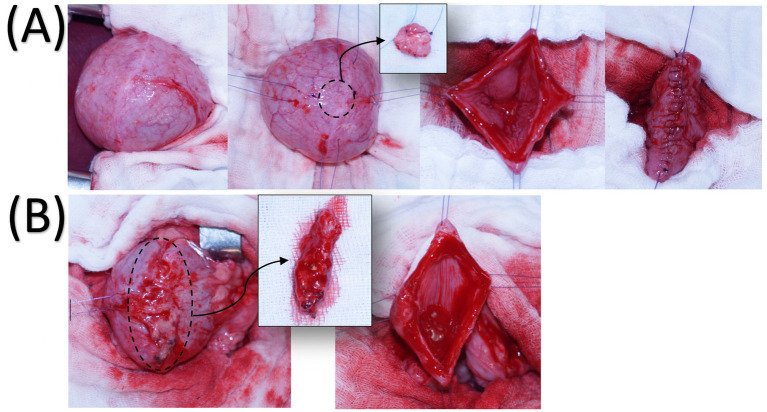
Full-thickness bladder biopsy **(A)**. After isolation of the apex of the bladder, stay sutures were placed and a biopsy of approximately 1 cm^3^ was obtained from the apex of the bladder. Bladder lumen and mucosa were examined for any abnormality and the bladder was closed with a single continuous suture. Biopsy of the bladder on day 10 **(B)**. The apex of the bladder is isolated and the scar of the previous biopsy identified. Four stay sutures are placed and the scar is excised. The biopsy is approximately 2 cm length by 0.5 cm width and 1 cm thick.

On day 0, serving as the baseline control time point, the dog underwent a full thickness biopsy of the bladder wall of approximately 1 cm^3^ under general anesthesia (same protocol as previously) to induce the acute bladder inflammatory (Figure 2A). The following day, as well as on days 4 and 8 after bladder biopsy, 20×10^6 autologous PKH26-ADMSCs were injected over 5 min in the cephalic vein. Each bolus was individually prepared for each injection, using previously frozen cells, which were thawed, cultured, and then labeled at their fifth passage, following the procedure described in the *in vitro* study. Each bolus of ADMSCs was prepared using a Thoma cell counting chamber. An excision of the bladder scar at the site of the previous bladder biopsy (Figure 2B) was performed on day 10 to assess the presence of labelled ADMSCs. The dog was monitored for potential adverse effects during and after each ADMSCs administration. Urinalysis and urine assessment for fluorescence were performed on day 5.

#### 2.2.3 Stromal/stem cells detection in bladder tissue and urine

Cryosections of 10 μm were performed on the bladder biopsies frozen in OCT [Leica (Wetzlar, Germany)], stained with DAPI, a nuclear staining [Vector laboratories (Newark, CA, USA)] and monitored under a fluorescence microscope. After urine centrifugation at 300 *g*, supernatant of urine was discarded and sediment was spread on a slide and observed under a fluorescence microscope.

## 3 Results

### 3.1 ADMSCs culture, characterization and labeling

**Figure 3:**
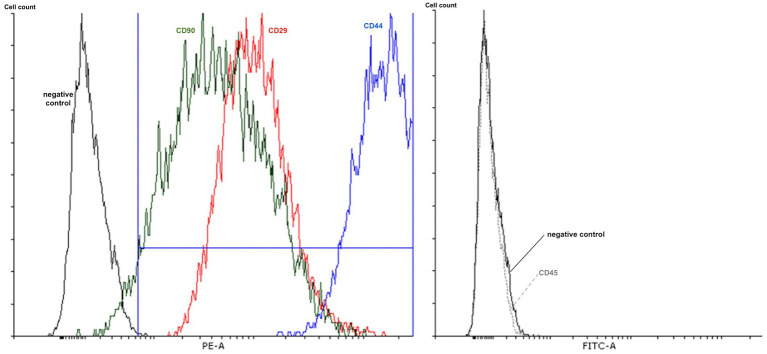
Adipose-derived mesenchymal stromal/stem cells characterization. X-axis, left: Phycoerythrin (PE) mean fluorescence intensity within the ADMSCs labelled with CD90 (green line), CD29 (red line) and CD44 (blue line) antibodies. X-axis, right: Fluorescein isothiocyanate (FITC)-A mean fluorescence intensity within CD45 (dashed line)-labelled ADMSCs. Black lines (left and right) correspond to negative controls. Cell count (y-axis, left and right).

**Figure 4:**
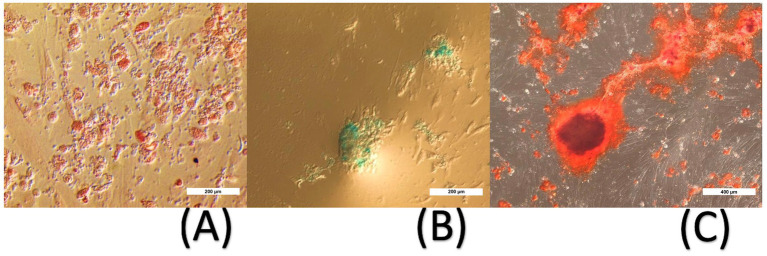
*In vitro* ADMSCs differentiation. Differentiation into adipogenic (**A**, O Red Oil, staining lipid droplets in red, 200x magnification), chondrogenic (**B**, Alcian blue, staining sulphated proteoglycans deposits in blue, 200x magnification,) and osteogenic (**C**, Alizarin red, showing a red colored matrix, 100x magnification) cells.

The *in vitro* culture method of 4-week duration allowed the harvesting, growing and long-term cryo-conservation of phenotypically characterized ADMSCs to enable 3 administrations of 20 × 10^6 cells each: mesenchymal stromal/stem cell positive markers were expressed (CD44, CD29 and CD90) while negative marker was not present (CD45) (Figure 3). The cells multipotency was demonstrated by their ability to differentiate into adipogenic, chondrogenic and osteogenic cells (Figure 4). PKH26-labelled canine ADMSCs still displayed 20% of fluorescence after 10 days *in vitro*.

### 3.2 Tracking of the ADMSCs

**Figure 5:**
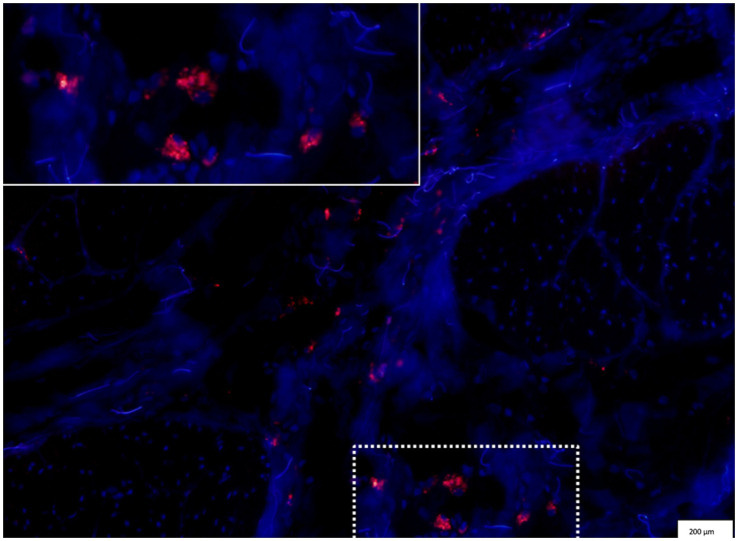
Red fluorescence of the PKH26-labelled ADMSCs in the target tissue. Fluorescence is identified within the bladder wall, in the scar of the previous biopsy (DAPI staining for nucleus localization, overlaid with the red fluorescent same picture to identify PKH26-labelled ADMSCs).

Within the bladder scar, autologous PKH26-labelled ADMSCs were identified at day 10. They appeared as cells overlaid by a fluorescent punctiform labelling (Figure 5). This finding confirmed that the administration technique and labelling were efficient and that ADMSCs engraftment into the bladder injury site was possible after intravenous injections. Fluorescence was not observed within the bladder wall on control time (day 0), neither in the urine when monitored on day 5.

### 3.3 Safety

During the whole study, the clinical exam of the dog (awareness, heart rate, respiratory rate, color of mucosal membranes, capillary refill time, pulse and abdominal palpation) was normal. The dog presented mild and self-limiting hematuria on days 2 and 5. Urinary tract ultrasonography on day 5 showed an increased thickness of the ventral bladder wall, with focal hyperechoic spots within the bladder wall. Free gas and focal steatitis were observed in the caudal abdomen. These abnormalities were compatible with the recent bladder biopsy and the presence of suture material. Complete cell blood count, blood urea nitrogen and plasma creatinine values were investigated on day 5 and were within normal limits. Urinalysis was also performed on day 5 and showed degenerated red blood cells with absence of fluorescent cells, bacteria, or inflammatory cells. Thus, hematuria was attributed to the recent bladder surgery and probably not related to the IV administration of ADMSCs.

Five years after completion of the study, the dog was still bright and alert and no abnormality was detected on routine thoracic radiographs, abdominal ultrasonography and electrocardiogram.

## 4 Discussion

The novelty of the present study phase relies on the autologous nature of the ADMSCs and their IV administration in several boluses in a dog. This species is gaining popularity in the field of regenerative therapy research. Indeed, conditions that naturally develop in dogs and cats and resemble diseases in humans such as osteoarthritis, inflammatory bowel disease and atopic dermatitis can be used as disease models for stem cell studies (43).

Intravenous administration of MSCs is reported to have lower efficiency and specificity compared to intra-arterial or parenchymal administration, but it is less invasive and consequently, easily transposable for clinical purposes (44). Moreover, a systemic injection not only mimics the migration route of the endogenous stem cells to the target site, but it also has the potential to improve the immunomodulatory effects of the MSCs (45). The main drawback of IV injection is pulmonary trapping, which reduces the number of cells available for the target organ. To optimize the pulmonary passage, more cells should be administered and several boluses are encouraged (46). Indeed, rats with myocardial infarction had better long term cardiac function when 4 injections of MSCs were performed compared to 2 injections (47). On the contrary, no significant benefit was observed for canine atopic dermatitis after a single IV injection of autologous ADMSCs (48). This led some authors to suggest that pulmonary trapping makes questionable the therapeutic benefit of stem cells after IV injection (46) while others point out the potential benefit for pulmonary diseases (49). However, reports of clinical benefit may be explained by the differences between cell lines that can display variable susceptibility to the adverse environment of blood stream and xenobarrier, as well as variable capacity to express integrins, that are necessary for cell diapedesis and parenchymal engraftment (44). Another explanation may be a phenomenon of redistribution of the cells to other organs after lung entrapment, due to homing of the MSCs (11).

Our objective was to assess the homing capability of autologous ADMSCs to a bladder lesion following IV injection, and not to confirm pulmonary and splenic trapping, since this phenomena have already been confirmed after three IV administrations of canine allogenic adipose-derived mesenchymal stem cells (50) in a dog with induced spinal cord injury. Therefore, pulmonary and splenic biopsies were not performed in the present study to prevent unnecessary morbidity. Our findings confirmed that IV boluses of autologous PKH26-labelled ADMSCs administered on days 1, 4 and 8 allowed to find these cells in a bladder injury created on day 0. We were also able to find these cells into the *linea alba* (surgical approach to access the bladder) of the same dog (data not shown). This supports the logic of systemic dilution of the ADMSCs towards any injured site after intravenous administration and justifies *a posteriori* our protocol of multiple IV injections of high dose of ADMSCs.

Mesenchymal stromal/stem cells IV administration necessitates their tracking to ascertain that they reach the target organ. We chose to use PKH26 as labelling tracker. It has been used on human mesenchymal stromal cells with no effect on proliferation and attachment (51). Cell labelling also enables quantification of the engraftment (52). Other methods of labelling have shown to rapidly decrease within 24 h post injection (53) and ideal timing for cells detection is unknown. It may vary depending on labelling agent and cell type, origin, age and other factors. In our study, we did not detect ADMSCs in the urine, but we may have missed them due to unrepeated and unregular checks following injection. Indeed, it was not the scope of the study to elucidate the future of the ADMSCs after engraftment, so our protocol did not include several urine inspections for fluorescence. In a study, cell labelling with PKH26 resulted in a significant reduction of detection during the first days post transplantation, to reach approximately 40% of fluorescence intensity at day 10 and less than 5% after 42 days (54). In another study, identification of PKH26-labelled MSCs in host tissues was still possible 14 days after transplantation (17). Since we did not perform sequential samples of the bladder, we were not able to monitor the evolution of the labelling marker throughout the 10 days of the study. However, being aware of the progressive reduction of labelling intensity and due to our different mode of administration (IV and not parenchymal), we chose a high frequency of ADMSCs administration in order to enhance our chance to catch their presence into the targeted bladder tissue at the termination of the experimental period. Thus, we confirmed that PKH26-labelled cells can still be observed in the target tissue 10 days after initial administration.

While cell labelling allowed us to validate our hypotheses regarding homing to the target tissue of autologous ADMSCs following IV administration, identifying cells into the targeted organ does not demonstrate that differentiation or paracrine activity will occur (55). In this study phase in one dog, we did not intent to assess the therapeutic efficiency of the ADMSCs. Rather, we were aware of any potential adverse reactions. Adverse reactions were not observed within the 10 days of the study period and until 5 years later. We noticed a mild self-limiting hematuria within the 10 days of the study period, most probably due to the surgical bladder biopsy that to the administration of PKH26 autologous ADMSCs. Adverse reactions have been reported after IV injection of allogenic or xenogenic stromal/stem cells. Sudden death was observed in mice after human stem cell administration, however this was attributed to accidental rapid injection as it was not observed with slow infusion (56). In dogs, reported adverse reactions are variable, which make unclear whether causality can directly be attributed to the MSCs, to the recipient individual or to any other step of the protocol. In a study on 9 healthy adult dogs receiving IV unlabeled allogenic bone-marrow derived MSCs at different dosages, vomiting, increased respiratory and heart rates and increased body temperature were observed in one dog. In the same study, histological changes in the lungs and variations in the white blood cell count were observed in 4 dogs, while electrocardiogram and coagulation status remained unchanged (20). In our study, we did not observe these changes during the physical examination of the dog, nor in its white blood cell count. In diseased dogs, pain and inflammation at the injection site were reported in one of 4 dogs after IV injection of allogenic adipose-derived mesenchymal stem cells (57), while this was not observed in our dog. In another study, several injections of allogenic adipose-derived mesenchymal stem cells in a puppy affected by canine parvovirus did not cause adverse reaction (58). Other studies have also reported the absence of adverse reaction after allogenic stem cell transplantation, whether injections were multiple (18) or single (53), whether the cells were labelled (53) or not (59), whether the dogs had a localized pathology (18, 19, 59) or were healthy (53). In our study, the absence of short-term systemic adverse reaction illustrates the safe use of PKH26 autologous ADMSCs through IV route in that dog, but it may also be related to the healthiness of the dog, so caution should be taken when considering specific conditions. Additionally, we obtain data about long term safety in the dog of the present study. Indeed, stem cells may affect chromosomal stability, as well as metastasis of existing tumors (60). While arrhythmogenicity and tumorigenesis have been advocated as potential long term adverse effects of MSCs use (61, 62), the dog of the present study was still healthy 5 years after the study: thoracic radiographs, abdominal ultrasonography and electrocardiogram did not show evidence of any abnormality.

Autologous administration of MSCs is recommended to reduce the risk of immune rejection by the host but also to reduce the risk of transferring infectious agents (63). While the risk of host rejection is of major interest when multiple injections are planned, allogenic MSCs are usually considered as immunoprivileged, but this necessitates to be tested in clinical trials of repeated injections (64). Cryopreservation of canine autologous adipose-derived mesenchymal stem cells of 12 months duration has been shown to preserve stemness features (65), which encourages autologous use. A benefit of using autologous MSCs may rely on clinical effect. In patients with ischemic cardiomyopathy, clinical improvement was preferentially observed in autologous versus allogenic group of patients receiving bone-marrow derived MSCs, while alloimmune reaction was observed in less than 5%, was mild and not acute (reported as an increase in panel reactive antibodies 6 months after stem cell administration) (66). However, this argument is contradicted by a review that reported no difference in the therapeutic efficacy between allogenic and autologous MSC administration (62). In our study, the advantages of using autologous versus allogenic stromal/stem cells were multiple: it allowed us to reduce the number of dogs to a single one (donor and host) while optimizing our chance to get results without any potential rejection of the cells, even with repeated administrations.

The main limitation of our study is the use of one single dog. We did not enroll a control dog with a sham injection because it would not have helped us to accept or reject our study hypothesis. We did not want to enroll many dogs as for a controlled study, being aware of potential debilitating side effects for the dogs or failure of ADMSCs homing. The present research is also a pilot study for a second study, in which the homing of autologous ADMSCs to a bladder injury after multiple IV injections will be a prerequisite. In this larger study, the emphasis will be put on the therapeutic effect of the ADMSCs, less on their homing and engraftment success. Other limitations encountered in previous studies have also been encountered in our protocol. Firstly, the choice of adipose tissue has an evident effect in the differentiation potential of the ADMSCs (67). We selected adipose tissue as the tissue of origin of MSCs because of its abundance throughout the body and ease of access when obtained from the subcutaneous fat (63) compared to visceral origin (68). Another interest of adipose origin (compared to bone marrow origin) is a potential better ability of the cells to maintain their undifferentiated state and to self-renew (40). Moreover, ADMSCs can be prepared more rapidly, compared with longer times of expansion and complicated isolation procedures if bone marrow or skeletal muscle are selected as donor sites of MSCs (60). Secondly, the optimal dose of MSCs, timing and method of delivery to obtain a therapeutic effect are unknown and may vary with species, tissues, and organs (45). For potential future clinical transposition, we elected for an IV administration. We chose a high dose of ADMSCs in each bolus, similar to IV dosages of 1,3 × 10^6^ cells per kilogram body weight (48) or 1 to 2 × 10^6^ cells per kilogram body weight (18) previously reported, and we performed early and frequent administrations of ADMSCs after the bladder injury. Finally, the objective of this pilot phase was not to study the bladder healing process in a single dog, nor to assess the future of the ADMSCs after engraftment. Therefore, we did not perform any specific analysis on the scar nor on the rest of the bladder.

## 5 Conclusion

PKH26-labeled ADMSCs were obtained from the subcutaneous adipose tissue of a healthy adult dog. Isolation and labelling of these cells did not affect their homing capacity. Indeed, after three IV injections of 20×10^6 cells each, the cells were detected in the bladder wall of the same dog at the level of a recent biopsy site. A recent inflammation, created by a tissue biopsy, is an effective stimulus to home the ADMSCs to the bladder, even after IV injection and the potential consecutive lung or spleen trapping. Whether homing is emphasized by the use of autologous cells remains uncertain.

While these findings will need to be confirmed on a larger number of dogs, the results of this pilot study suggest that the use of autologous MSCs and their administration through an IV route could be considered in a larger population of dogs to pursue investigations on stromal/stem cell effects after engraftment at the injured target site.

## Data Availability

The original contributions presented in the study are included in the article/supplementary material, further inquiries can be directed to the corresponding author.
